# Determination of a Robust Assay for Human Sperm Membrane Potential Analysis

**DOI:** 10.3389/fcell.2019.00101

**Published:** 2019-06-11

**Authors:** Carolina Baro Graf, Carla Ritagliati, Cintia Stival, Paula A. Balestrini, Mariano G. Buffone, Darío Krapf

**Affiliations:** ^1^CONICET-UNR, Laboratoty of Cell Signal Transduction Networks, Instituto de Biología Molecular y Celular de Rosario, Rosario, Argentina; ^2^UNR, Laboratorio de Medicina Reproductiva, Facultad de Ciencias Bioquímicas y Farmacéuticas, Rosario, Argentina; ^3^Consejo Nacional de Investigaciones Científicas y Tecnológicas, Instituto de Biología y Medicina Experimental, Buenos Aires, Argentina

**Keywords:** membrane potential, population fluorimetry, sperm capacitation, human sperm, carbocyanine dye, diSC3(5)

## Abstract

Mammalian sperm must undergo a complex process called capacitation in order to fertilize the egg. During this process, hyperpolarization of the sperm plasma membrane has been mostly studied in mouse, and associated to its importance in the preparation to undergo the acrosome reaction (AR). However, despite the increasing evidence of membrane hyperpolarization in human sperm capacitation, no reliable techniques have been set up for its determination. In this report we describe human sperm membrane potential (*Em*) measurements by a fluorimetric population assay, establishing optimal conditions for *Em* determination. In addition, we have conducted parallel measurements of the same human sperm samples by flow cytometry and population fluorimetry, before and after capacitation, to conclusively address their reliability. This integrative analysis sets the basis for the study of *Em* in human sperm allowing future work aiming to understand its role in human sperm capacitation.

## Introduction

During fertilization, the sperm cell has a simple and fundamental goal: to merge with the oocyte and deliver its genetic material. However, mammalian sperm are not able to fertilize an oocyte shortly after ejaculation. To gain fertilizing competence, they must go through a process called sperm capacitation. Capacitation includes a complex series of molecular events that normally take place in the female genital tract but can be mimicked *in vitro* in defined media. This process prepares sperm to acquire hyperactivated motility (HA) and to undergo the acrosome reaction (AR) upon stimulation (Chang, 1951; Austin, 1952).

At the molecular level, sperm capacitation is associated with increased membrane fluidity, changes in intracellular ion concentrations (Visconti et al., 2011), hyperpolarization of the sperm plasma membrane (Zeng et al., 1995; Hernández-González et al., 2006), increased activity of protein kinase A (PKA) (Krapf et al., 2010), and protein tyrosine phosphorylation (Arcelay et al., 2008). Data regarding capacitation has been acquired in different mammalian species. Moreover, due to the complexity of the capacitation process, each of these events has been studied independently. Thus, knowledge regarding how these events interconnect to regulate capacitation is mostly unavailable.

One important molecular process leading to the capacitated state is hyperpolarization of the sperm plasma potential, which has been mostly studied in mouse (Zeng et al., 1995; Hernández-González et al., 2006; De La Vega-Beltran et al., 2012). In this species, *Em* hyperpolarization has been shown to be both necessary and sufficient for sperm to undergo stimulated AR (De La Vega-Beltran et al., 2012). The mechanisms that drive *Em* hyperpolarization in mouse sperm involve the opening of the potassium channel Slo3 (Chávez et al., 2013), since sperm from Slo3 KO mice fail to hyperpolarize during capacitation. Most importantly, these sperm do not undergo acrosomal reaction upon stimulation (Santi et al., 2010; Yang et al., 2011), and, as expected from these data, Slo3 KO male mice are sterile. Despite the clear importance of membrane hyperpolarization during mouse sperm capacitation, this *Em* change has not been studied in detail in human sperm. A few reports have independently analyzed *Em* of sperm samples: while Linares-Hernández et al. (1998) reported an *Em* for non-capacitated sperm of around −40 mV, Patrat et al. (2005) reported that capacitated sperm exhibit an *Em* of about −58 mV. Both studies used fluorimetric population assays using the carbocyanine DiSC_3_(5). In addition, a qualitative study suggested by flow cytometry that human sperm undergo hyperpolarization during capacitation (Brewis et al., 2000). It is worth noticing that the consortium of Drs. Treviño and Darszon, further substantiated these qualitative findings, showing by flow cytometry that a subpopulation of human sperm hyperpolarizes during capacitation (López-González et al., 2014). However, these studies did not measure *Em* changes quantitatively on the same sample, i.e., during capacitation, which is crucial for proper human sperm analysis considering the biological variabilities found in these cells.

Despite the clear importance of hyperpolarization in mouse sperm cells, data regarding human sperm is scarce. Our manuscript presents an integrative study, since the same samples have been evaluated through flow cytometry analysis and population *Em* measurements, both before and after capacitation. We have established a solid reproducible methodology. Moreover, our data show that hyperpolarization is observed in human sperm along incubation in capacitating conditions, although alternative behaviors are also reported here. Finally, the methodology and implications of *Em* hyperpolarization in human sperm are herein discussed.

## Materials and Methods

### Reagents

Chemicals were obtained from the following sources: bovine serum albumin (BSA) and HEPES were purchased from Sigma (St. Louis, MO, United States). Propidium iodide (PI) from Santa Cruz (Santa Cruz, CA, United States), 3,3-dipropylthiadicarbocyanine iodide [DiSC_3_(5)] from Invitrogen (Carlsbad, United States). All other chemicals were of analytical grade.

### Ethical Statement

The study protocol was approved by the Bioethics Committee of the Instituto de Biología y Medicina Experimental (CONICET). All subjects gave written informed consent in accordance with the Declaration of Helsinki.

### Culture Media

HEPES-buffered human tubal fluid (HTF) was used throughout the study, containing (in mM) 4.7 KCl, 0.3 KH_2_PO_2_, 90.7 NaCl, 41.2 MgSO_4_, 2.8 Glucose, 1.6 CaCl_2_, 3.4 Sodium Piruvate, 60 Sodium Lactate, 23.8 HEPES, and was termed non-capacitating media. For capacitating media, 15 mM NaHCO_3_, and 0.5% w/v BSA were added. In all cases, pH was adjusted to 7.4 with NaOH.

### Human Sperm Preparation

Semen samples were obtained by masturbation from 10 healthy donors after 3–5 days of abstinence and analyzed following WHO recommendations (World Health Organization, 2010). All samples fulfilled semen parameters (total fluid volume, sperm concentration, motility, viability, and morphology) according to WHO normality criteria. Samples were allowed to liquefy for 1 h at 37°C in water bath. Then, sperm ejaculates were allowed to swim-up in non-capacitating HTF media (see above) at 37°C for 1 h. Motile selected spermatozoa were washed 5 min 400 × *g*. Conditions were performed in 400 μl of non-capacitating or capacitating media to a final cell concentration of 7 × 10^6^ cells/ml for fluorimetric population assays and 1.25 × 10^6^ cells/ml for flow cytometry measurements. Sperm were capacitated for 3 h at 37°C.

### Determination of Membrane Potential by Flow Cytometry

Sperm plasma membrane potential (*Em*) was assessed using different concentrations of DiSC_3_(5) for 10 min, for proper determination of dye concentration. Unless specified, dye concentration was then of 50 nM. In addition, 2 μM of PI was added 30 s before collecting data to monitor viability. Data were recorded as individual cellular events using a BD FACSAria^TM^ II cell sorter flow cytometer (BD Biosciences). Forward scatter (FSC) and side scatter (SSC) fluorescence data were collected from 20,000 events per sample. Debris and cell aggregates were excluded from analysis by gating side vs. FSC cytogram. Negative stain for PI was selected for living cells and positive cells for DiSC_3_(5) were detected using the filter for allophycocyanine (APC) (660/20). Normalization was performed by adding 1 μM valinomycin and 39.6 mM KCl. Data were analyzed using FACS Diva and FlowJo software (Tree Star 10.0.7r2).

### Determination of Membrane Potential by Fluorimetric Population Assay

After incubation in each condition, human sperm, at a concentration of 3 × 10^6^ sperm in 1.7 ml, were loaded with DiSC_3_(5) at different concentrations to determine the ideal dye concentration. Unless specified, dye concentration was determined at 1 μM (dissolved in DMSO at 5 mM). Sperm were transferred to a gently stirred cuvette at 37°C, and the fluorescence was monitored with a Varian Cary Eclipse fluorescence spectrophotometer at 620/670 nm excitation/emission wavelengths. Recordings were initiated when steady-state fluorescence was reached (approximately 10 min). Calibration was performed by adding 1 μM valinomycin and sequential additions of KCl (in μL): 4, 4, 7, and 15 for calibration curve 1; 8, 13.34, 13.1, and 13.3 for calibration curve 2; 1.67, 3.4, 5.95, and 10.2 for calibration curve 3. Unless specified, calibration curve 1 was used. Final sperm membrane potentials were obtained by linearly interpolating the theoretical *Em* values against arbitrary fluorescence units of each trace. The theoretical *Em* values were obtained using Nernst equation, considering 120 mM the internal K^+^ concentration in sperm. This internal calibration for each determination compensates for variables that influence the absolute fluorescence values.

### Statistical Analysis

One-way repeated measures analysis of variance was used to compare between *Em* values obtained with different calibration curves. Statistical significances are indicated in the figure legends.

## Results

### *Em* Measurement in Human Sperm

**FIGURE 1:**
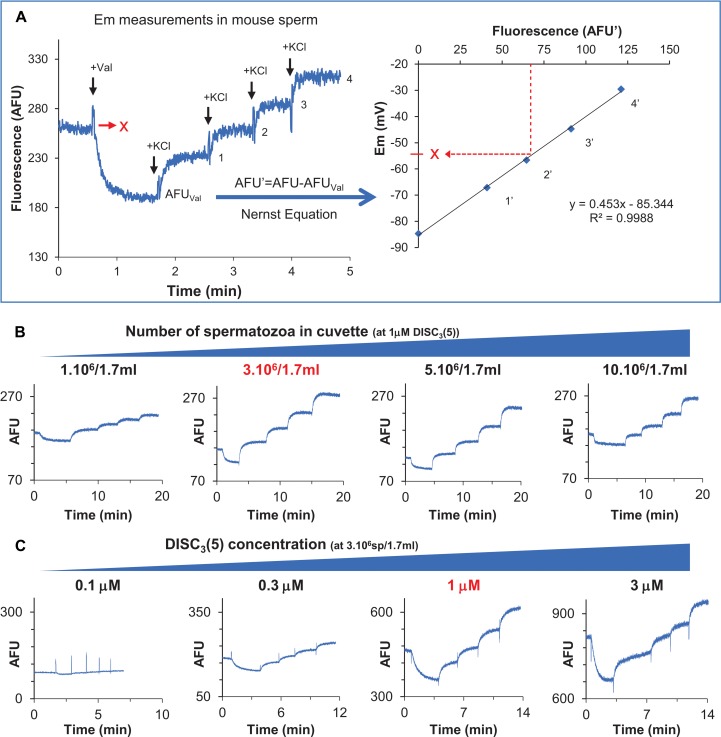
Optimization of a population fluorimetry assay to determine human sperm membrane potential (*Em*). **(A)** *Em* measurement in mouse sperm. Left panel shows a representative trace, indicating valinomycin, and KCl additions. Right panel corresponds to the resulting calibration curve obtained after calculating *Em* for each fluorescence value using the Nernst equation. The initial fluorescence value is then interpolated to reach the resting membrane potential value. **(B)** Representative fluorescence traces using different human sperm concentrations at 1 μM DiSC_3_(5). Optimal number of sperm is indicated in red. **(C)** Representative fluorescence traces using different dye concentrations, at constant sperm number (3 × 10^6^/1.7 ml). Optimal dye concentration is indicated in red.

*Em* measurements were performed with a positively charged carbocyanine probe, DiSC_3_(5). This dye partitions into sperm cells according to their membrane potential but independently on the nature of ionic fluxes, rendering it suitable for potential measurements of the plasma membrane. The technique has long been used in mouse sperm, in a robust and reproducible way, as shown in Figure 1A (Chou et al., 1989; Zeng et al., 1995; Demarco et al., 2003). The dye is added to the sperm suspension, where it partitions into the cells originating a decrease in fluorescence due to accumulation of the dye in the lipid bilayer of the cells, in a less-fluorescent form (Plášek and Hrouda, 1991). *Em* hyperpolarization favors the influx of more dye to the cell, resulting in decreased extracellular fluorescence. Once the dye gives a stable signal, reached after a 10 min incubation in the sperm suspension, calibration starts by adding valinomycin (a K^+^ ionophore) followed by sequential additions of KCl, increasing extracellular K^+^ concentration to different known values. The original resting potential can be obtained following the Nernst equation, provided that the membrane behaves as a K^+^ electrode where the internal K^+^ concentration is 120 mM (Darszon et al., 1999; Figures 1A,B).

At appropriate concentrations of sperm and probe, this method provides a highly reproducible value of plasma membrane potential. In order to analyze optimal conditions for human sperm, both sperm and probe concentrations were assayed. When the probe was fixed at 1 μM, shifting sperm cell number from 1 × 10^6^ to 1 × 10^7^ (in a cuvette containing 1.7 ml) gave different responses. The best dynamic range was observed when 3 × 10^6^ sperm were used (1.76 × 10^6^ sperm/ml) (Figure 1C). Once the optimal sperm number was determined, different DiSC_3_(5) concentrations were tested. As seen in Figure 1C, no other concentration improved signal to noise ratio when compared to 1 μM DiSC_3_(5). Accordingly, 1 μM dye and 3 × 10^6^ sperm/1.7 ml where taken as optimal conditions for the subsequent analysis of human sperm *Em*. Even though DiSC_3_(5) bears a short alkyl group that renders it poorly hydrophobic, migration to the mitochondria cannot be totally excluded, which would originate minor contributions to the observed *Em*. However, as showed in Supplementary Figure 1, the mitochondrial electron transport inhibitor rotenone did not affect DiSC_3_(5) signal. Thus, *Em* measurements of both non capacitated and capacitated sperm were unaffected by mitochondrial dysfunction, further substatntiating that mitochondrial contribution is negligible.

### Calibration of *Em* Measurements

**FIGURE 2:**
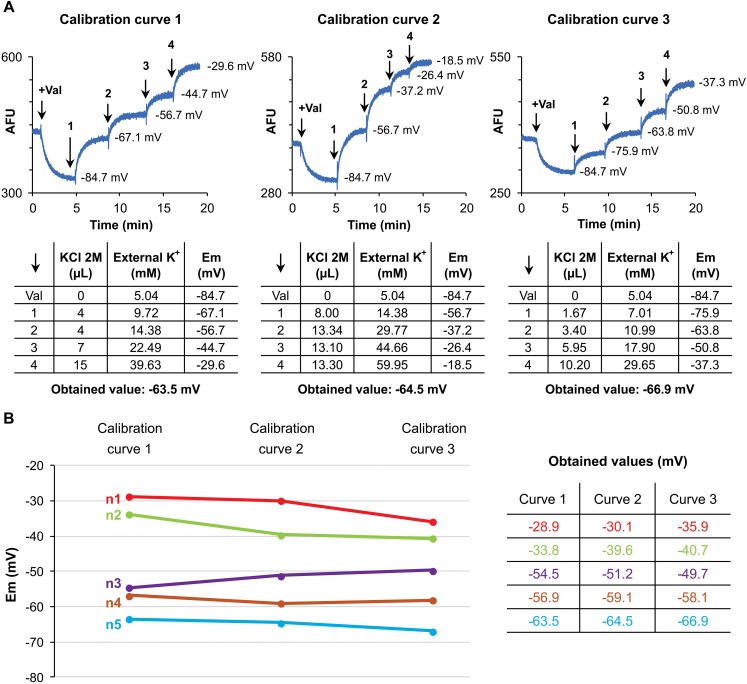
Calibration of *Em* measurements. **(A)** Upper panels show fluorescence traces for each calibration curve tested (1–3), indicating *Em* values after sequential KCl additions. Lower panels show KCl volume added, external K^+^ concentration and *Em* obtained for each addition. Finally, the resting membrane potential of each measurement is indicated. **(B)** Membrane potential values obtained after testing the described calibration curves in five different samples. No significant differences were observed, *p* > 0.05.

In order to avoid errors due to dye loading or sperm concentration differences, every measurement carries an internal calibration curve after fluorescence stabilization (Ritagliati et al., 2018). As shown in Figure 2A, calibration was performed by adding 1 μM valinomycin and sequential additions of KCl. Initial potassium concentration equals 5.04 mM KCl; then, additions of a 2 M KCl solution, sequentially rose KCl concentration to 9.72, 14.38, 22.49, and 39.63 mM, corresponding to *Em* of −84.7, −67.1, −56.7, −44.7, and −29.6 mV, respectively. With the aim of assessing the strength of this method applied to human sperm samples, three different calibration curves were performed (Figure 2A, curves 1–3), varying the concentration of KCl added. In the examples shown in Figure 2A, the resting membrane potentials were of −63.5, −64.5, and −66.9 mV for calibration curves 1–3, respectively. This procedure was performed on 5 different sperm samples. As shown in Figure 2B, the technique showed no significant variations among different calibration curves, supporting the strength of the methodology.

### Comparative Analysis Between Flow Cytometry and Fluorometric Population Assays

Up to now, the few studies that have reported *Em* measurements in human sperm have mostly used flow cytometry, while only a couple of them have used the fluorescent population assay, in either capacitated or non-capacitated sperm. However, quantitative measurements of *Em* during capacitation is clearly missing. Thus, we decided to analyze human sperm *Em* during capacitation, using both flow cytometry and population fluorimetry approaches.

**FIGURE 3:**
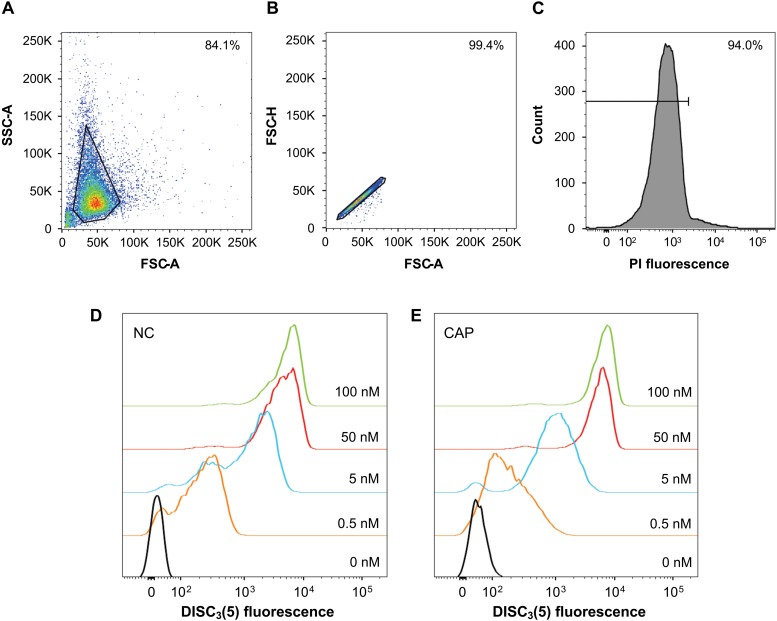
Optimization of a flow cytometry assay to determine human sperm membrane potential. **(A)** Dot plot of side (SSC-A) vs. forward (FSC-A) scatter for each sample. Region of interest to eliminate cellular debris is shown. **(B)** The forward scatter height (FSC-H) vs. forward scatter area (FSC-A) profile was used to exclude cell aggregates and large cells from the analysis. **(C)** Histogram of PI fluorescence where live cells (low stain) were selected. **(D)** Human sperm were incubated with different dye concentrations in non-capacitating (left panel) and **(E)** capacitating conditions (right panel) by flow cytometry to determine optimal loading conditions.

Firstly, in order to establish appropriate working conditions, sperm were loaded with 50 nM DiSC_3_(5) and run on a flow cytometer. A plot of cell complexity (SSC-A) vs. cell size (FSC-A) is shown in Figure 3A, detailing the gating used to eliminate cell debris and aggregates. The selection of individual cells was confirmed using the FSC-H vs. FSC-A plot (Figure 3B). Live cells were selected after co-staining sperm with PI, normally obtaining a cellular viability >90% (Figure 3C). This representative set up was performed after loading cells with different DiSC_3_(5) concentrations (0.5–100 nM), for both non-capacitated (Figure 3D) and capacitated sperm (Figure 3E). From these data, 50 nM DiSC_3_(5) resulted the concentration of choice, being the lowest concentration tested with highest intracellular fluorescence. Final cell concentration was fixed to 1.25 × 10^6^ sperm/ml.

**FIGURE 4:**
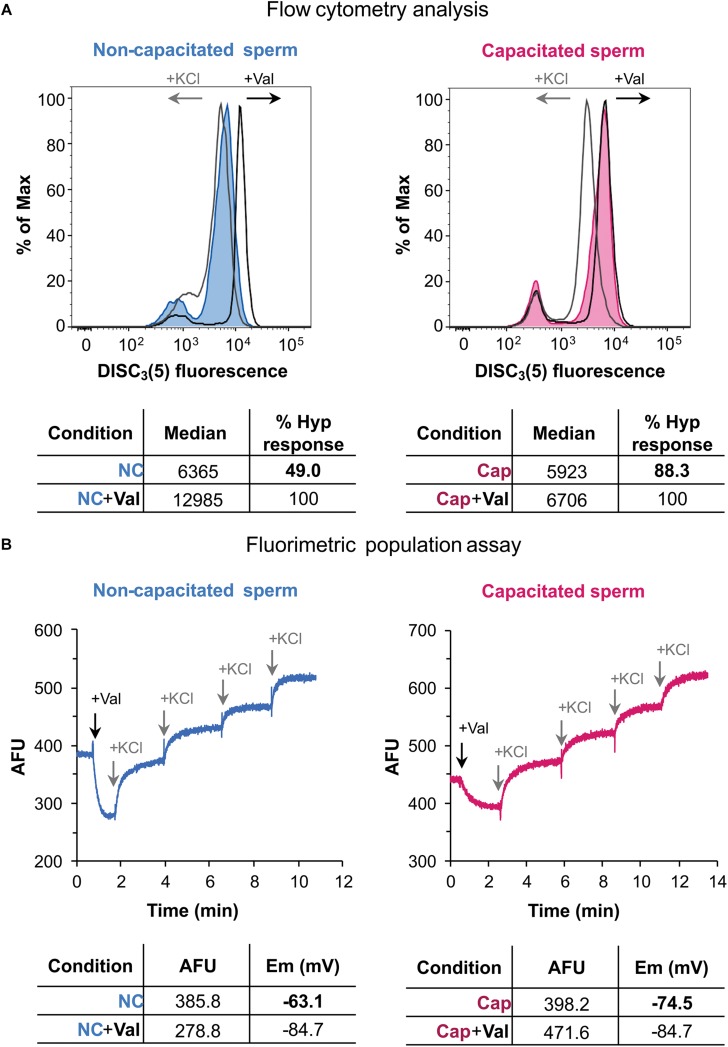
Human sperm membrane potential determination by flow cytometry and population fluorimetry. **(A)** Flow cytometry analysis of human sperm in non-capacitating (left panel) and capacitating conditions (right panel). Valinomycin addition causes an increase of fluorescence, in accordance to a hyperpolarized state; KCl addition causes a decrease of fluorescence, as expected for a depolarized state. Each histogram plots percentage of maximum (% of Max) vs. DiSC3(5) fluorescence, obtained by normalizing to the peak height at the mode of the distribution – so the maximum *Y*-axis value in the absolute-count histogram becomes 100% of total. Lower panel shows median values obtained before and after valinomycin addition. Hyperpolarization response is normalized against valinomycin addition. **(B)** Population fluorimetric assay of human sperm in non-capacitating (left panel) and capacitating conditions (right panel). In this case valinomycin addition causes a decrease in fluorescence, as expected for a hyperpolarized state; KCl sequential additions increase extracellular fluorescence, depolarizing the sample. Lower panel shows fluorescence values for initial and hyperpolarized states, with their corresponding membrane potential value (in mV). These results are representative of one experiment (*n* = 5).

Secondly, these conditions were used to analyze *Em* by flow cytometry, as shown in Figure 4A. In flow cytometry using DiSC_3_(5) (a cationic dye), increased fluorescence correlates to *Em* hyperpolarization since more dye enters the cell. Addition of valinomycin further increases fluorescence, as the sperm cells hyperpolarize (Figure 4A). Subsequent addition of KCl caused the expected depolarization of the sperm cells, rendering lower values of fluorescence. When a capacitated sperm sample was analyzed, valinomycin only caused a slight increase in fluorescence (Figure 4A). However, KCl highly depolarized these hyperpolarized cells. Thus, it could be inferred from these data, that this sample suffered hyperpolarization during capacitation.

In order to compare *Em* measurements of two samples by flow cytometry, the median fluorescence was obtained after valinomycin addition, corresponding to the maximum hyperpolarization state. The median value from the initial state (before valinomycin addition) of each sample can then be used to calculate a normalized degree of hyperpolarization (Figure 4A). Accordingly, in non-capacitating conditions the hyperpolarization degree was 49% shifting to 88.3% in capacitating conditions. This normalization is necessary not only for a semi-quantitative analysis, but also when performing a qualitative comparison between conditions (i.e., NC and CAP), since dye concentrations could differ among them.

When the same sperm sample incubated under either non-capacitating or capacitating conditions were analyzed by fluorimetry, values of −63.1 and −74.5 mV were obtained, supporting the data obtained by flow cytometry (Figure 4B). It is worth noticing that occasionally, approximately 10% of samples, exhibited unexpected behaviors when analyzed by flow cytometry: upon addition of valinomycin, instead of causing hyperpolarization, a shift to a depolarized state was observed (Supplementary Figure 2). When these samples were analyzed by fluorimetry, it was observed that, as expected, valinomycin caused an initial hyperpolarization that is, however, immediately followed by a sustained depolarization (Supplementary Figure 2). This result indicates that what was observed as a valinomycin-triggered depolarization, was in fact a later compensation by still unknown ion fluxes, and further supporting the strength of fluorimetric measurements.

## Discussion

In mouse sperm, changes in the sperm plasma membrane potential (*Em*) associated with sperm capacitation have been studied for more than 20 years (Zeng et al., 1995). Cauda epidydimal mouse sperm have a depolarized *Em* of ∼−40 mV, which hyperpolarizes to ∼ −60 mV when capacitation takes place reviewed by Stival et al. (2016). These values of mouse sperm *Em* have been conducted using fluorimetric measurements and constitute an average value of a population known to be far from homogenous. Thus, when other techniques as single cell microscopy (Arnoult et al., 1999) or flow cytometry (López-González et al., 2014; Escoffier et al., 2015) were used, two clear sperm sub-populations displaying different *Em* were observed. One of these sub-populations remains at a depolarized state, while the other shifts to a relatively hyperpolarized value of *Em* (∼−80 mV) (Arnoult et al., 1999). These findings are consistent with the observation that only a fraction of the sperm cells are capable to undergo the AR (Salicioni et al., 2007).

In human sperm, it has been recently proposed that certain *Em* shift is associated with normal sperm function, as assessed by electrophysiology and IVF outcome (Brown et al., 2016). However, this study involved laborious techniques that hampered the analysis of many cells per patient. Other studies involved the qualitative analysis of *Em* in human sperm by flow cytometry (Brewis et al., 2000) and analysis of either non-capacitated or capacitated sperm by fluorimetry (Linares-Hernández et al., 1998; Patrat et al., 2005). Considering the importance of the study of *Em* in human sperm using straightforward techniques, we aimed to standardize a reliable method for the analysis of *Em* during human sperm capacitation.

We have satisfactorily established the optimal conditions to quantitatively measure human sperm *Em* using the carbocyanine DiSC_3_(5) with a fluorimeter in a population assay. But most importantly, we have compared two different methods, i.e., flow cytometry and population fluorimetry, by using the same sperm sample. Our data indicate that *Em* analysis by flow cytometry is a robust and reproducible methodology, when proper normalization for loading control is performed. However, it is important to highlight results that, although very reproducible, are to date difficult to understand. After addition of valinomycin, some samples experience an initial hyperpolarization that is followed by a depolarization. These behaviors are easily followed by fluorimetry, and can then be analyzed accordingly. However, when cytometry is used, due to technical time limitations, depolarization is observed instead of the expected valinomycin driven hyperpolarization. Thus, even though both techniques give the same end point result, cytometry might omit this particular behavior, and give a confusing result.

The mechanisms that regulate the hyperpolarization of human sperm plasma membrane during capacitation are poorly understood. Plasma membrane permeability to the ionic media at any given time defines the cell *Em*. Thus, *Em* changes during capacitation reflect changes of ion permeability. The most relevant ions are Na^+^, K^+^, and Cl^–^, which have an equilibrium potential of +40, −80, and −40 mV, respectively. Considering these ion’s equilibrium, closure of Na^+^ transport as well as opening of a K^+^ channel could drive hyperpolarization. In mouse, the current knowledge points toward a high contribution of K^+^ to this phenomenon. However, the situation is far from clear in human sperm. Re-depolarization might in turn be associated to opening of Na^+^ channels and/or Cl^–^ channels, shifting the equilibrium to more positive values. The methodology herein described is ideal for standardizing a technique that could aid in the study of human *Em* physiology.

## Data Availability

All datasets generated for this study are included in the manuscript and/or the Supplementary Files.

## Ethics Statement

The study protocol was approved by the Bioethics Committee of the Instituto de Biologa y Medicina Experimental (CONICET). All subjects gave written informed consent in accordance with the Declaration of Helsinki.

## Author Contributions

CBG, CR, CS, and PB conducted the experiments. All authors analyzed the data and revised final version of the manuscript. CBG, CR, MGB, and DK conceived the study. CBG, CR, and DK wrote the manuscript.

## Conflict of Interest Statement

The authors declare that the research was conducted in the absence of any commercial or financial relationships that could be construed as a potential conflict of interest.
