# Supplementary figures and images for: Determination of a Robust Assay for Human Sperm Membrane Potential Analysis
Source: Front Cell Dev Biol. 2019 Jun 11;7:101. doi: 10.3389/fcell.2019.00101 (PMC6579818; doi:10.3389/fcell.2019.00101)

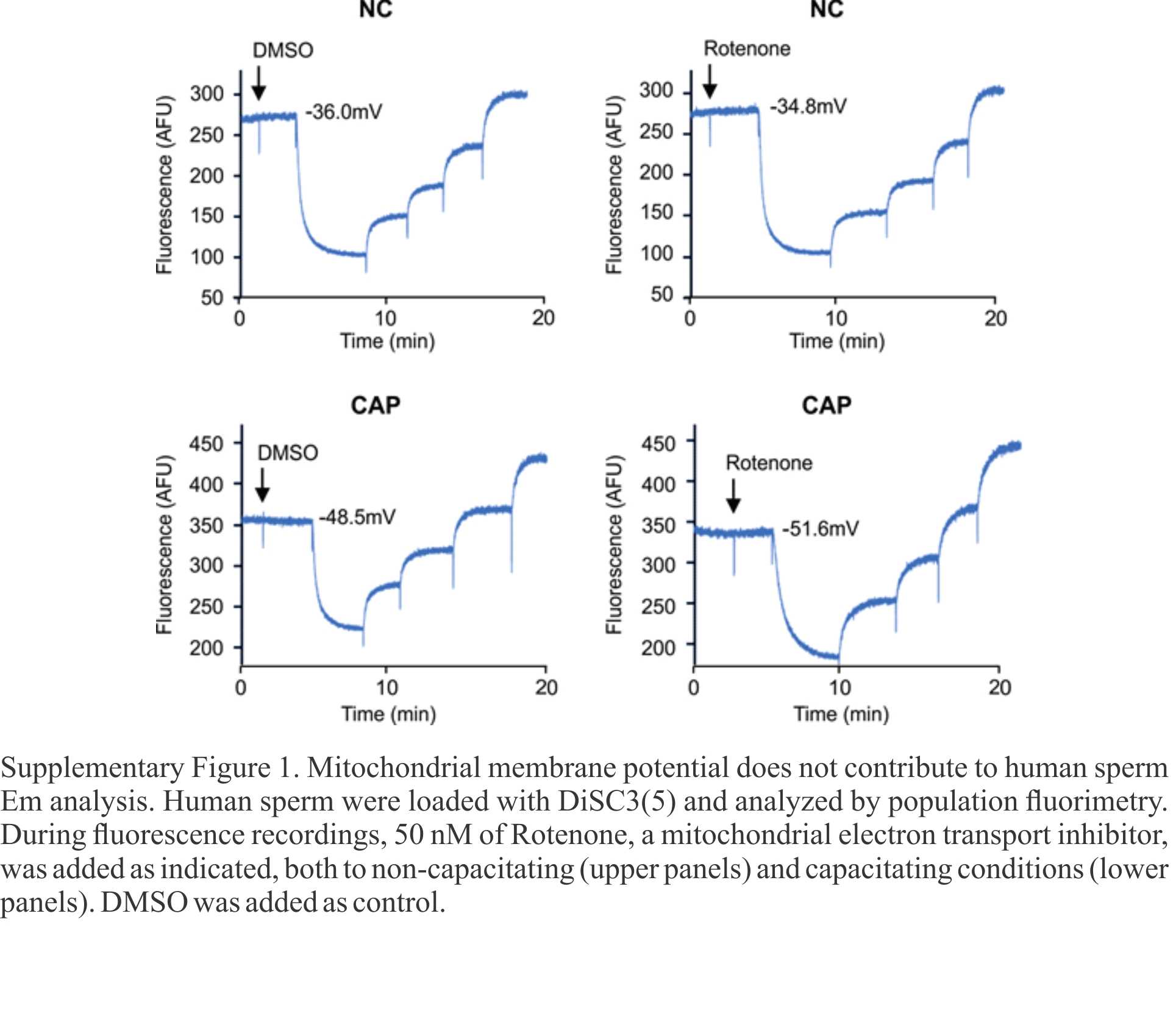

Supplement: Supplementary file 1 [file Image_1.JPEG]

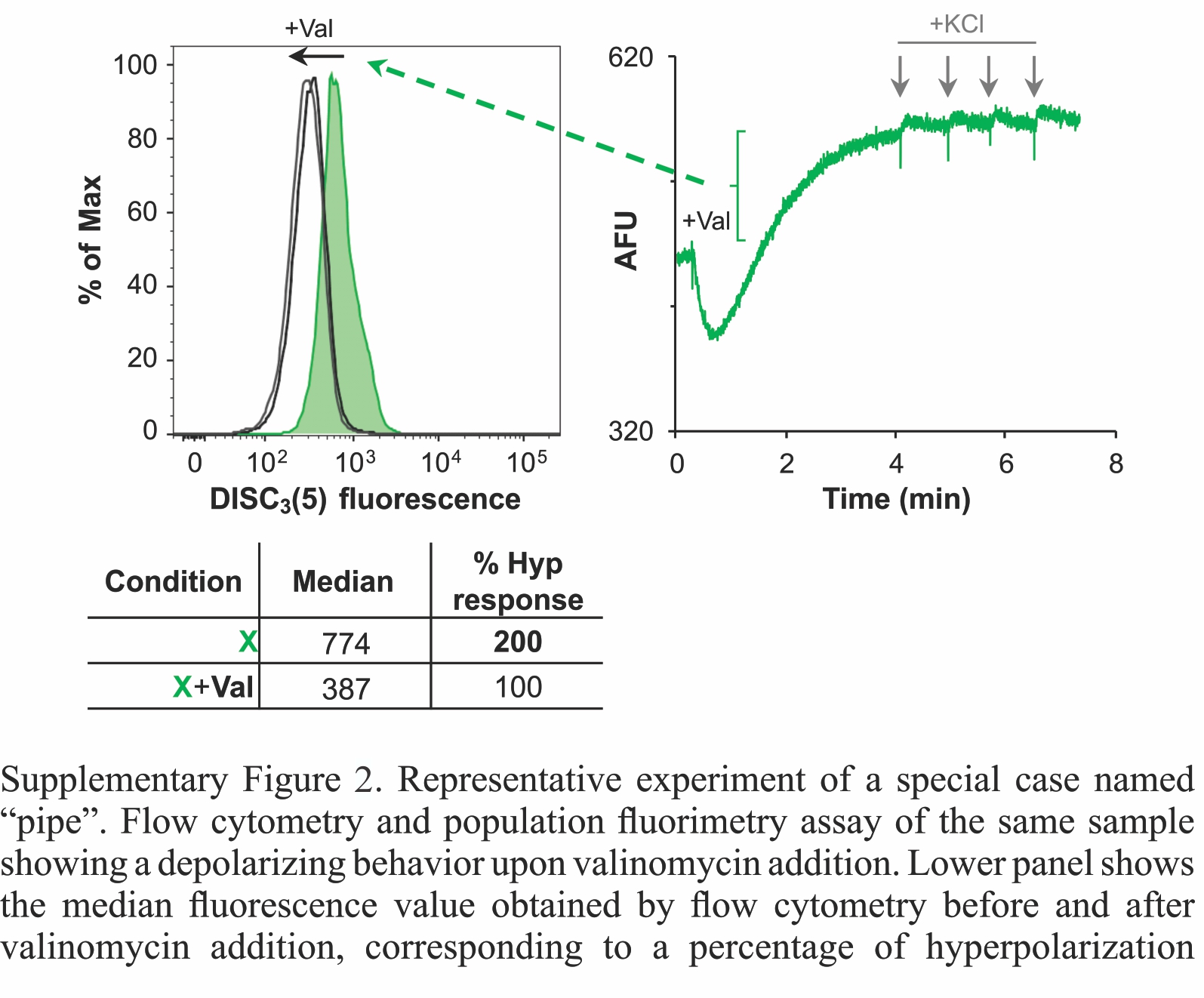

Supplement: Supplementary file 2 [file Image_2.JPEG]
